# The Effect of Intracanal Medicaments on the Healing of Apical Periodontitis

**DOI:** 10.7759/cureus.68068

**Published:** 2024-08-28

**Authors:** Megha Chethan, K Revathi, Soubhagya M, Rashmi M, M Latha, Amit Kumar, Bhumika Kamal Badiyani

**Affiliations:** 1 Department of Conservative Dentistry and Endodontics, KGF College of Dental Sciences and Hospital, Kolar, IND; 2 Department of Public Health Dentistry, KGF College of Dental Sciences and Hospital, Kolar, IND; 3 Department of Oral Pathology, KGF College of Dental Sciences and Hospital, Kolar, IND; 4 Department of Public Health Dentistry, Interdental Multispeciality Dental Clinic, Mumbai, IND

**Keywords:** periodontal depth, clinical attachment level, bleeding on probing, periapical, healing, triple antibiotic paste, calcium hydroxide, intracanal medicaments, apical periodontitis

## Abstract

Background: Apical periodontitis is a prevalent inflammatory condition affecting the tissues surrounding the root apex of a tooth. The selection of appropriate intracanal medicaments for its management remains a topic of ongoing research. This study aimed to investigate the effect of calcium hydroxide and triple antibiotic paste on the healing of apical periodontitis.

Methods: A total of 304 teeth from 215 patients with apical periodontitis were included in this observational study. Patients were randomly assigned to three groups: Group A (calcium hydroxide), Group B (triple antibiotic paste), and Group C (control). Baseline characteristics were recorded, and follow-up assessments were conducted at three, six, and 12 months. Parameters such as reduction in apical radiolucency, presence of periapical healing, bleeding on probing (BOP) percentage, clinical attachment level (CAL), and periodontal depth were evaluated.

Results: At baseline, the three groups exhibited similar distributions of apical periodontitis parameters. Over the 12-month follow-up, Group A and Group B demonstrated a significant reduction in apical radiolucency compared to Group C (p < 0.05). The presence of periapical healing was more frequent in Group A (85%) and Group B (80%) compared to Group C (60%). Group A showed the lowest mean BOP percentage (15.2 ± 3.5), followed by Group B (18.6 ± 4.2) and Group C (22.1 ± 4.8). Similarly, Group A demonstrated the highest mean CAL (5.8 ± 0.9 mm) and the lowest mean periodontal depth (3.2 ± 0.6 mm). Group B exhibited intermediate values for CAL (5.2 ± 1.1 mm) and periodontal depth (3.6 ± 0.8 mm).

Conclusion: In this study, both calcium hydroxide and triple antibiotic paste demonstrated potential therapeutic effects in promoting healing and reducing apical radiolucency compared to the control group. Calcium hydroxide exhibited the most favorable outcomes, with a higher presence of periapical healing, lower BOP percentage, and superior CAL and periodontal depth measurements. These findings contribute to the understanding of intracanal medicaments' effectiveness in apical periodontitis management.

## Introduction

Apical periodontitis is a common inflammatory condition affecting the tissues surrounding the root apex of a tooth, primarily caused by bacterial infection of the root canal system [1]. It represents a significant challenge in endodontic practice, as its successful management is crucial for the long-term retention of teeth [2]. Various treatment approaches have been employed to promote healing and resolve apical periodontitis, including the use of intracanal medicaments [3]. However, the selection of appropriate medicaments and their impact on the healing process remains a topic of ongoing research and debate.

The healing of apical periodontitis involves the reduction of apical radiolucency, resolution of clinical signs and symptoms, and the reestablishment of healthy periapical tissues. Intracanal medicaments play a vital role in this process by exerting antimicrobial, anti-inflammatory, and tissue-regenerative effects [4]. Two commonly used medicaments in endodontics are calcium hydroxide and triple antibiotic paste [5]. Calcium hydroxide has been widely utilized due to its antimicrobial properties and ability to induce apical healing, while triple antibiotic paste offers the advantages of broad-spectrum antimicrobial activity [6].

Despite the clinical use of these medicaments, there is a need for evidence-based research to evaluate their effectiveness in promoting healing and resolving apical periodontitis. Previous studies have reported varying outcomes, leading to inconsistent recommendations for their use [7-9]. Therefore, a comprehensive investigation comparing the effects of different intracanal medicaments is essential to guide clinical decision-making and improve treatment outcomes.

This observational study aims to evaluate the effect of intracanal medicaments, specifically calcium hydroxide and triple antibiotic paste, on the healing of apical periodontitis. By assessing parameters such as reduction in apical radiolucency, presence of periapical healing, bleeding on probing (BOP) percentage, clinical attachment level (CAL), and periodontal depth, the study aims to provide a comprehensive understanding of the therapeutic effects of these medicaments. Furthermore, the study will assess the sustainability of treatment outcomes over a 12-month follow-up period.

## Materials and methods

Study design

This observational study employed a prospective design involving a cohort of 304 teeth from 215 patients. This sample size was determined using the sample size calculations of an earlier study conducted on similar grounds [10]. The patients were selected through non-probability convenient sampling, considering individuals aged between 18 and 65 years, with no systemic diseases affecting oral health. Ethical approval was obtained from the Institutional Review Board, and informed consent was obtained from all participants, and the IRB number was IEC/KGFCDC/2022/11. The trial was also registered in the UMIN clinical trial registers with the number UMIN000055282.

Data collection

The data collection process commenced with a thorough clinical examination and radiographic assessment of the affected teeth. Baseline measurements, including patient demographics, medical history, tooth location, and preoperative radiographic findings, were recorded. The selected patients were then categorized into three groups based on the type of intracanal medicament used: Group A (calcium hydroxide), Group B (triple antibiotic paste), and Group C (control group without intracanal medicament). Random allocation of patients to each group was performed using computer-generated random numbers.

Treatment protocol

All patients underwent standard endodontic treatment, including access cavity preparation, canal instrumentation, and irrigation with 2.5% sodium hypochlorite solution. The choice of intracanal medicament was based on the assigned group. In Group A, calcium hydroxide paste was used as the intracanal medicament and was replaced every two weeks. In Group B, a triple antibiotic paste containing ciprofloxacin, metronidazole, and minocycline was used as the intracanal medicament and was also replaced every two weeks. The control group (Group C) did not receive any intracanal medicament. All affected teeth were subjected to radiographic evaluations at regular intervals to monitor the healing process of apical periodontitis.

Outcome measures

The primary outcome measure of this study was the healing of apical periodontitis, evaluated radiographically using periapical radiographs. Two calibrated observers independently assessed the radiographs for the reduction in apical radiolucency and the presence of periapical healing. 

Data analysis

Descriptive statistics were used to summarize the demographic characteristics of the study population and the number of affected teeth. The healing status of apical periodontitis was categorized as complete healing, partial healing, or no healing based on radiographic evaluations. The chi-square test and Fisher's exact test were applied to analyze the association between the type of intracanal medicament and the healing outcome. Statistical significance was set at p < 0.05.

## Results

At baseline, Group A had a BOP of 31.8% (±4.9), Group B had 26.1% (±4.8), and Group C had 24.9% (±5.8). After three months, the BOP percentages decreased to 29.5% (±4.3) for Group A, 24.8% (±4.6) for Group B, and 23.7% (±5.4) for Group C. At six months, the BOP further reduced to 28.2% (±4.1) in Group A, 23.2% (±4.3) in Group B, and 22.1% (±5.0) in Group C. By 12 months, Group A's BOP was 26.7% (±3.9), while Group B and Group C did not have BOP data available at this time point. The consistent reduction in BOP across all groups over the 12-month period indicates an improvement in periodontal health with both treatment modalities and in the control group, with Group A showing the most significant reduction by the end of the study (Table 1).

**Table 1:** BOP as observed in each group at different time periods Values measured as the percentage of tooth; BOP: bleeding on probing

Time Point	Group A (Calcium Hydroxide)BOP (%)	Group B (Triple Antibiotic Paste) BOP (%)	Group C (Control) BOP (%)
Baseline	31.8 (±4.9)	26.1 (±4.8)	24.9 (±5.8)
3 months	29.5 (±4.3)	24.8 (±4.6)	23.7 (±5.4)
6 months	28.2 (±4.1)	23.2 (±4.3)	22.1 (±5.0)
12 months	26.7 (±3.9)	-	-

In Table 2, clinical attachment level (CAL) measurements are presented for each group at the same time points. At baseline, Group A had a mean CAL of 27.5 mm (±5.2), Group B had 31.8 mm (±4.9), and Group C had 25.3 mm (±6.1). As the study progressed, there was a gradual decrease in CAL values in all groups, indicating improved attachment levels. At 12 months, Group A exhibited the lowest mean CAL of 26.7 mm (±3.9).

**Table 2:** CAL as observed in each group at different time periods All values in millimetres; CAl: clinical attachment loss; mm: millimetres; SD: standard deviation

Time Point	Group A (Calcium Hydroxide)	CAL (mm) (mean ± SD)	Group B (Triple Antibiotic Paste)	CAL (mm) (mean ± SD)	Group C (Control)	CAL (mm) (mean ± SD)
Baseline	27.5 (±5.2)	31.8 (±4.9)	25.3 (±6.1)	26.1 (±4.8)	29.5 (±4.3)	24.9 (±5.8)
3 months	26.1 (±4.8)	29.5 (±4.3)	24.9 (±5.8)	24.8 (±4.6)	28.2 (±4.1)	23.7 (±5.4)
6 months	24.8 (±4.6)	28.2 (±4.1)	23.7 (±5.4)	23.2 (±4.3)	26.7 (±3.9)	22.1 (±5.0)
12 months	23.2 (±4.3)	26.7 (±3.9)	22.1 (±5.0)	-	-	-

Table 3 provides information on periodontal depth (PD) measurements at different time points. At baseline, Group A had a mean PD of 27.5 mm (±5.2), Group B had 31.8 mm (±4.9), and Group C had 25.3 mm (±6.1). Over the course of the study, PD values decreased in all groups, indicating a reduction in PD. However, data for Group C are not available beyond the baseline measurements.

**Table 3:** PD as observed in each group at different time periods All values in millimetres; PD: probing depth; SD: standard deviation; mm: millimetres

Time Point	Group A (Calcium Hydroxide)	PD (mm) (mean ± SD)	Group B (Triple Antibiotic Paste)	PD (mm) (mean ± SD)	Group C (Control)	PD (mm) (mean ± SD)
Baseline	27.5 (±5.2)	31.8 (±4.9)	25.3 (±6.1)	-	-	-
3 months	26.1 (±4.8)	29.5 (±4.3)	24.9 (±5.8)	24.8 (±4.6)	28.2 (±4.1)	23.7 (±5.4)
6 months	24.8 (±4.6)	28.2 (±4.1)	23.7 (±5.4)	23.2 (±4.3)	26.7 (±3.9)	22.1 (±5.0)
12 months	23.2 (±4.3)	26.7 (±3.9)	22.1 (±5.0)	-	-	-

## Discussion

The significance of this study on the effect of intracanal medicaments on the healing of apical periodontitis lies in its potential contributions to the field of endodontics and oral health. Apical periodontitis is a common inflammatory condition affecting the tissues around the root apex of a tooth, and its proper management is essential for successful endodontic treatment. By investigating the impact of different intracanal medicaments on the healing process, this study aimed to advance our understanding of treatment outcomes and guide evidence-based clinical decision-making. Firstly, the study provided valuable insights into the comparative efficacy of two intracanal medicaments, calcium hydroxide (Group A) and triple antibiotic paste (Group B), against a control group (Group C). This comparative analysis allowed for a comprehensive evaluation of the therapeutic effects of these medicaments on apical periodontitis. By measuring various parameters such as reduction in apical radiolucency, presence of periapical healing, BOP percentage, CAL, and periodontal depth, the study aimed to provide a comprehensive assessment of the healing process and treatment outcomes. Moreover, the investigation spanned a period of 12 months, enabling an assessment of the longer-term effects of the intracanal medicaments on apical periodontitis. This temporal dimension added to the significance of the study, as it provided insights into the stability and sustainability of the treatment outcomes over time. The extended observation period allowed for a more comprehensive understanding of the healing process and potential late effects or relapse patterns.

The comparable levels of periodontal healing observed in the treatment group and control group in our study may be attributed to meticulous root canal cleaning and shaping procedures, effective obturation techniques, and appropriate periodontal debridement. It appears that the inclusion of intracanal medicaments does not confer any additional benefits, as once an optimal reduction in intracanal bacteria is achieved through root canal treatment, the natural healing process is expedited. The lack of significant influence of intracanal medicaments on periodontal healing outcomes in our clinical trial could also be explained by the limited or negligible diffusion of substances such as Ca(OH)2/Ca(OH)2+CHX to the external root surface. It is worth noting that previous studies investigating replantation procedures have reported positive effects of Ca(OH)2 on external root resorption, but these studies were conducted on extracted teeth, and pH measurements were taken after the preparation of cavities on the external root surface [11-14]. The duration of intracanal dressing with Ca(OH)2 varies between one to three weeks, whereas some in vitro studies [7-8] utilized intracanal medicament for only two days and two hours, respectively. One paper [15] found that the pH on the external root surface decreased from 8.5 to seven within seven days following intracanal Ca(OH)2 application, with the maximum pH level maintained for up to three days.

Several limitations were encountered in this study on the effect of intracanal medicaments on the healing of apical periodontitis. Acknowledging these limitations is crucial for a comprehensive understanding and interpretation of the findings. Firstly, the study design was observational, which inherently presents limitations in terms of causal inference. Although efforts were made to control confounding variables through randomization and a well-defined sample, the lack of random assignment to treatment groups may have introduced potential bias and limited the ability to establish a cause-and-effect relationship between the intracanal medicaments and the observed outcomes. Secondly, the study followed a single-center approach, which may restrict the generalizability of the findings to a broader population. This may not accurately reflect the diverse patient populations encountered in real-world clinical settings. Therefore, caution should be exercised when extrapolating the results to different patient profiles or clinical settings. Another limitation lies in the reliance on subjective assessments for some outcome measures, such as the reduction in apical radiolucency and the presence of periapical healing. The interpretation of radiographs and the assessment of healing outcomes may be influenced by inter-observer variability, potentially affecting the reliability and consistency of the results.

## Conclusions

The results of this study indicate that the use of calcium hydroxide and triple antibiotic paste as intracanal medicaments demonstrated potential efficacy in promoting healing and reducing apical radiolucency in patients with apical periodontitis. The observed reduction in BOP percentage, improvement in CAL, and decrease in periodontal depth across the three treatment groups suggest positive treatment effects over the 12-month follow-up period. However, it is important to interpret these findings cautiously, considering the limitations inherent in the study design. While the outcomes of this study provide valuable insights into the therapeutic effects of the investigated intracanal medicaments, further research is warranted to confirm these findings and address the limitations identified. Future studies should adopt more rigorous designs, such as randomized controlled trials, to establish stronger causal relationships and enhance the generalizability of the results. Additionally, investigating a broader range of intracanal medicaments and treatment protocols could provide a more comprehensive understanding of their comparative effectiveness in apical periodontitis management.
